# Supplementary material for: Meta-Analysis and Gene Set Enrichment Relative to ER Status Reveal Elevated Activity of MYC and E2F in the “Basal” Breast Cancer Subgroup
Source: PLoS One. 2009 Mar 9;4(3):e4710. doi: 10.1371/journal.pone.0004710 (PMC2650420; doi:10.1371/journal.pone.0004710)
Supplement: Dataset S1 — GSEA results from first screen. The file contains a zipped archive of the HTML results of the second GSEA screen. Reports for enriched or depleted datasets may be accessed from the “index.html” link within the my_analysis.GseaPreranked.1219975950625.rpt.zip ZIP archive. Enrichment plots are available for each of the 50 most enriched and depleted gene sets. (13.32 MB ZIP) [file pone.0004710.s001.zip › my_analysis.GseaPreranked.1219975950625.rpt.zip!//GOLDRATH_CELLCYCLE.html]

Details for gene set GOLDRATH\_CELLCYCLE[GSEA]

|  || Dataset | zSco.ERneg.to.ERpos\_collapsed |
| Phenotype | NoPhenotypeAvailable |
| Upregulated in class | na\_pos |
| GeneSet | GOLDRATH\_CELLCYCLE |
| Enrichment Score (ES) | 0.75109285 |
| Normalized Enrichment Score (NES) | 2.3914268 |
| Nominal p-value | 0.0 |
| FDR q-value | 0.0 |
| FWER p-Value | 0.0 |
Table: GSEA Results Summary

  

Fig 1: Enrichment plot: GOLDRATH\_CELLCYCLE      
 Profile of the Running ES Score & Positions of GeneSet Members on the Rank Ordered List

  

| PROBE | GENE SYMBOL | GENE\_TITLE | RANK IN GENE LIST | RANK METRIC SCORE | RUNNING ES | CORE ENRICHMENT || 1 | BUB1 |  |  | 41 | 6.445 | 0.0590 | Yes |
| 2 | CCNA2 |  |  | 52 | 6.297 | 0.1188 | Yes |
| 3 | AURKB |  |  | 76 | 6.038 | 0.1752 | Yes |
| 4 | CDC45L |  |  | 108 | 5.824 | 0.2290 | Yes |
| 5 | GMNN |  |  | 206 | 5.302 | 0.2727 | Yes |
| 6 | CKS2 |  |  | 239 | 5.145 | 0.3198 | Yes |
| 7 | CHEK1 |  |  | 253 | 5.088 | 0.3678 | Yes |
| 8 | NCAPH |  |  | 348 | 4.799 | 0.4070 | Yes |
| 9 | CKS1B |  |  | 416 | 4.638 | 0.4466 | Yes |
| 10 | CCNB2 |  |  | 431 | 4.589 | 0.4897 | Yes |
| 11 | AURKA |  |  | 460 | 4.483 | 0.5308 | Yes |
| 12 | MAD2L1 |  |  | 477 | 4.446 | 0.5723 | Yes |
| 13 | PLK4 |  |  | 524 | 4.292 | 0.6102 | Yes |
| 14 | TACC3 |  |  | 669 | 3.995 | 0.6378 | Yes |
| 15 | S100A6 |  |  | 880 | 3.653 | 0.6572 | Yes |
| 16 | FYN |  |  | 952 | 3.549 | 0.6860 | Yes |
| 17 | CHAF1A |  |  | 971 | 3.525 | 0.7186 | Yes |
| 18 | PLK1 |  |  | 988 | 3.499 | 0.7511 | Yes |
| 19 | NEK2 |  |  | 1703 | 2.758 | 0.7239 | No |
| 20 | ECT2 |  |  | 2026 | 2.509 | 0.7239 | No |
| 21 | CDKN2C |  |  | 3148 | 1.866 | 0.6575 | No |
| 22 | CDC6 |  |  | 3185 | 1.847 | 0.6726 | No |
| 23 | CCNF |  |  | 3265 | 1.809 | 0.6841 | No |
| 24 | MAPK6 |  |  | 3396 | 1.751 | 0.6911 | No |
| 25 | RAD21 |  |  | 3642 | 1.642 | 0.6885 | No |
| 26 | INCENP |  |  | 5907 | 0.845 | 0.5264 | No |
| 27 | DUSP1 |  |  | 12483 | -3.240 | 0.0630 | No |
Table: GSEA details [plain text format]

  

Fig 2: GOLDRATH\_CELLCYCLE: Random ES distribution      
 Gene set null distribution of ES for **GOLDRATH\_CELLCYCLE**
